# Supplementary material for: Causal Relationship between Aging and Anorexia Nervosa: A White-Matter-Microstructure-Mediated Mendelian Randomization Analysis
Source: Biomedicines. 2024 Aug 16;12(8):1874. doi: 10.3390/biomedicines12081874 (PMC11351342; doi:10.3390/biomedicines12081874)
Supplement: Supplementary file 1 [file biomedicines-12-01874-s001.zip › Supplementary Figure/Figure S4/Singe SNP plot of MR analysis for TL to ubm-b-1503.pdf]

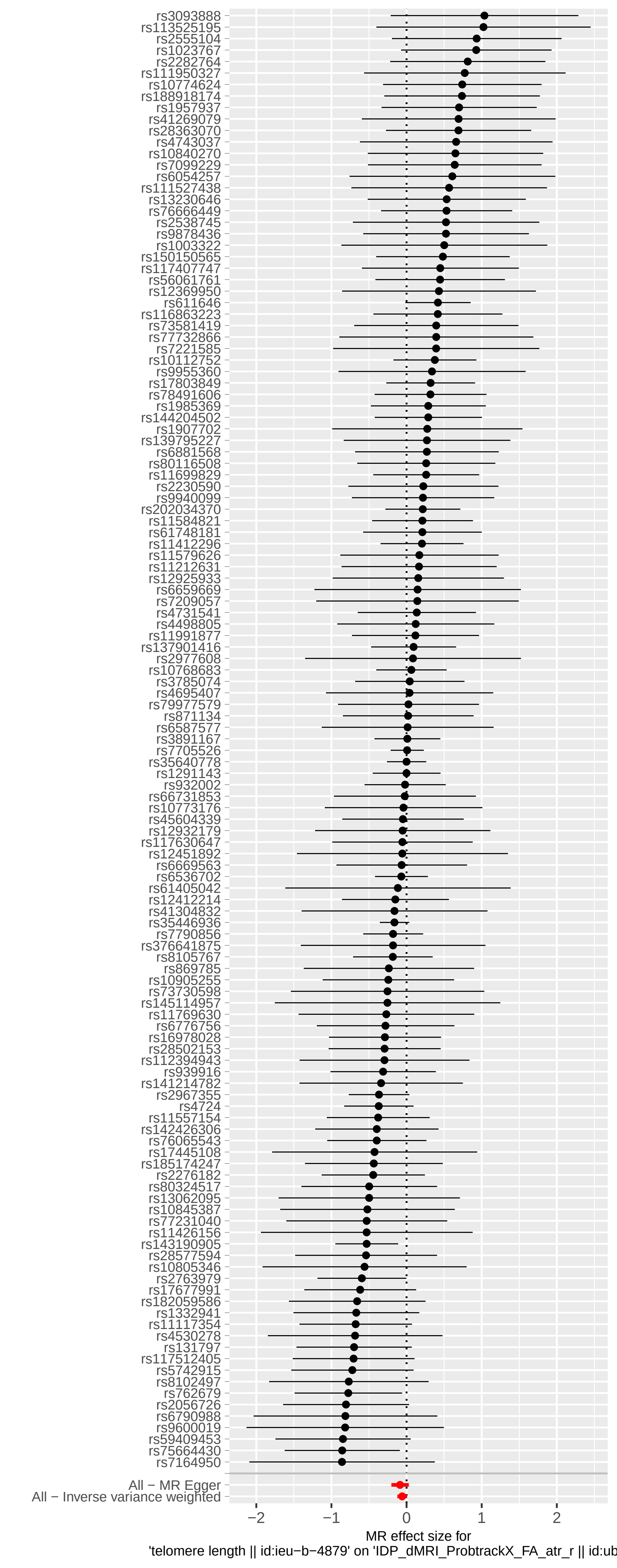
rs3093888
rs113525195
rs2555104
rs1023767
rs2282764
rs111950327
rs10774624
rs188918174
rs1957937
rs41269079
rs28363070
rs4743037
rs10840270
rs7099229
rs6054257
rs111527438
rs13230646
rs76666449
rs2538745
rs9878436
rs1003322
rs150150565
rs117407747
rs56061761
rs12369950
rs611646
rs116863223
rs73581419
rs77732866
rs7221585
rs10112752
rs9955360
rs17803849
rs78491606
rs1985369
rs144204502
rs1907702
rs139795227
rs6881568
rs80116508
rs11699829
rs2230590
rs9940099
rs202034370
rs11584821
rs61748181
rs11412296
rs11579626
rs11212631
rs12925933
rs6659669
rs7209057
rs4731541
rs4498805
rs11991877
rs137901416
rs2977608
rs10768683
rs3785074
rs4695407
rs79977579
rs871134
rs6587577
rs3891167
rs7705526
rs35640778
rs1291143
rs932002
rs66731853
rs10773176
rs45604339
rs12932179
rs117630647
rs12451892
rs6669563
rs6536702
rs61405042
rs12412214
rs41304832
rs35446936
rs7790856
rs376641875
rs8105767
rs869785
rs10905255
rs73730598
rs145114957
rs11769630
rs6776756
rs16978028
rs28502153
rs112394943
rs939916
rs141214782
rs2967355
rs4724
rs11557154
rs142426306
rs76065543
rs17445108
rs185174247
rs2276182
rs80324517
rs13062095
rs10845387
rs77231040
rs11426156
rs143190905
rs28577594
rs10805346
rs2763979
rs17677991
rs182059586
rs1332941
rs11117354
rs4530278
rs131797
rs117512405
rs5742915
rs8102497
rs762679
rs2056726
rs6790988
rs9600019
rs59409453
rs75664430
rs7164950
All − MR Egger
All − Inverse variance weighted
−2
−1
0
1
2
MR effect size for
'telomere length || id:ieu−b−4879' on 'IDP_dMRI_ProbtrackX_FA_atr_r || id:ub
